# Phenolic Compounds in the Potato and Its Byproducts: An Overview

**DOI:** 10.3390/ijms17060835

**Published:** 2016-05-27

**Authors:** Hazal Akyol, Ylenia Riciputi, Esra Capanoglu, Maria Fiorenza Caboni, Vito Verardo

**Affiliations:** 1 Department of Food Engineering, Faculty of Chemical and Metallurgical Engineering, Istanbul Technical University, Ayazağa Campus, Maslak, Istanbul 34469, Turkey; hazalakyol@gmail.com (H.A.); capanogl@itu.edu.tr (E.C.); 2 Department of Agro-Food Sciences and Technologies, Alma Mater Studiorum—University of Bologna, Piazza Goidanich 60, Cesena (FC) I-47521, Italy; ylenia.riciputi@unibo.it; 3 Inter-Departmental Centre for Agri-Food Industrial Research (CIRI Agroalimentare), University of Bologna, Piazza Goidanich 60, Cesena (FC) I-47521, Italy; 4 Department of Chemistry and Physics (Analytical Chemistry Area) University of Almería, Carretera de Sacramento s/n Almería E-04120, Spain; vito.verardo@unibo.it; 5 Research Centre for Agricultural and Food Biotechnology (BITAL), Agrifood Campus of International Excellence, ceiA3, University of Almería, Carretera de Sacramento s/n Almería E-04120, Spain

**Keywords:** *Solanum tuberosum*, potato, phenolic compounds, health effects

## Abstract

The potato (*Solanum tuberosum* L.) is a tuber that is largely used for food and is a source of different bioactive compounds such as starch, dietary fiber, amino acids, minerals, vitamins, and phenolic compounds. Phenolic compounds are synthetized by the potato plant as a protection response from bacteria, fungi, viruses, and insects. Several works showed that these potato compounds exhibited health-promoting effects in humans. However, the use of the potato in the food industry submits this vegetable to different processes that can alter the phenolic content. Moreover, many of these compounds with high bioactivity are located in the potato’s skin, and so are eliminated as waste. In this review the most recent articles dealing with phenolic compounds in the potato and potato byproducts, along with the effects of harvesting, post-harvest, and technological processes, have been reviewed. Briefly, the phenolic composition, main extraction, and determination methods have been described. In addition, the “alternative” food uses and healthy properties of potato phenolic compounds have been addressed.

## 1. Introduction

The potato (*Solanum tuberosum*) was known outside the Andes four centuries ago and has turned into a necessary component of much of the world’s cuisine. Following rice, wheat, and maize, it is the fourth largest food crop in the world and is very important for human consumption [1]. It was estimated that the total world potato production was 368 million tons in 2013; of that, 112 million tons were produced in the European Union [2].

This staple crop contains essential amino acids, vitamins, and minerals, and is thus reported to play a significant role in human nutrition [3]. Many varieties of potatoes offer nutritional quantities of ascorbic acid (up to 42 mg/100 g), potassium (up to 693.8 mg/100 g), dietary fiber (up to 3.3%), and other healthy bioactive components, with lesser amounts of protein (0.85%–4.2%) [4]. In particular, “resistant starch,” the dietary fiber that escapes digestion and absorption in the small intestine, is fermented by microorganisms in the large bowel [5]. Moreover, in a study it has been found that boiled potatoes showed the highest SI (satiety index) score out of 38 foods grouped into six food classes (fruit, bakery products, snack foods, carbohydrate-rich foods, protein-rich foods, and breakfast cereals) [6]. In addition to that, the potato is an ideal source of antioxidants for the human diet [7]. Despite the growing interest, little information has been reported about the important phytochemicals present in this widely consumed vegetable and its processing byproducts [8].

The worldwide utilization of potatoes is moving from fresh to processed potato products such as mashed and canned potatoes, fries, chips, and ready meals [9,10]. This industrial processing of potatoes creates huge amounts of peel as a byproduct and this generates disposal, sanitation, and environmental problems like all other industrial waste. Also, because of legal restrictions, the disposal of this waste poses a challenge [11,12]. However, potato peels are a great source of phenolic compounds because almost 50% of phenolics are located in the peel and adjoining tissues [8,13].

On the other hand, there is great interest in the utilization of natural antioxidants as functional ingredients in food formulation because they guarantee the cell constituents’ protection against oxidative damage and limit the risk of degenerative diseases linked to oxidative stress [14,15]. For these reasons, the use of these byproducts for the production of food ingredients with high nutritional value increased and, consequently, their recovery may be economically attractive [11,16].

If a global health goal is to expand the amounts of phytonutrients consumed in the diet, a valuable approach could be to improve the nutritional content of the phytochemicals in the most consumed crops, and/or use the bioactive compounds that are contained in vegetable waste [17]. The use of potato peel is underscored as a source of natural antioxidants by many previous studies [9,18,19]. Consequently, these bioactive compounds can be added in functional food or can be used to generate nutraceuticals by virtue of their potential health benefits.

## 2. Phenolic Compounds in Potatoes

Phenolic compounds are secondary metabolites produced in plants that have a common structure based on an aromatic ring with one or more hydroxyl substituents [20,21,22]. These compounds can be divided according to their chemical structure into flavonoids, phenolic acids, tannins, stilbenes, coumarins, and lignans [23,24]. Their presence affects the sensory qualities of plant-derived processed foods, including taste, color, and texture [25,26,27].

Potatoes are good sources of phenolic compounds, with total phenolic content higher than other widespread fruits and vegetables like carrots, onions, or tomatoes because of their high consumption rates [28]. The germplasm of the potato shows a striking variety in terms of the phenolic compounds profile and content [29]. The phenolic compounds are present in the potato peel and flesh; however, the peel is reported to have the highest amounts [30]. Phenolic compounds present in potatoes are phenolic acids and flavonoids including flavonols, flavanols, and anthocyanins [3].

### 2.1. Phenolic Acids

The predominant phenolic acids in plants are substituted derivatives of hydroxybenzoic acids and hydroxycinnamic acids. Caffeic, *p*-coumaric, and ferulic acids are the most common hydroxycinnamic acids and frequently occur in foods as esters with quinic acid or sugars [31]; hydroxybenzoic acid derivatives are mainly present in foods in the glucoside forms, and *p*-hydroxybenzoic, vanillic, and protocatechuic acids are the most common forms [31,32,33,34,35].

Phenolic acids are the most abundant phenolic compounds in potatoes [9,36,37,38]. Among all these phenolic acids, chlorogenic acid, which is the ester of caffeic acid and quinic acid, has been considerably described in potatoes [12,13,18,39,40]. It constitutes 90% of the phenolic compounds in potato peels [37,41] and exists in the form of three main isomers, chlorogenic acid (5-*O*-caffeoylquinic acid), neochlorogenic acid (3-*O*-caffeoylquinic acid), and cryptochlorogenic acid (4-*O-*caffeoylquinic acid) [42]. Also, caffeic acid is quantified at 25–72 mg/100 g in potatoes by many researchers [13,43,44,45].

Other phenolic acids such as ferulic acid, gallic acid, and *p*-coumaric acid have also been quantified in potatoes, ranging from 0 to 5 mg/100 g dry weight [8,9,36,46]; also, syringic acid, vanillic acid, sinapic acid, and salicylic acid are present in small quantities [3,9,31,38].

### 2.2. Flavonoids

Flavonoids represent the most common group of plant phenolic compounds and their presence influences the flavor and color of fruits and vegetables. The six significant subclasses of flavonoids are the flavones, flavonols, flavanones, flavan-3-ols, anthocyanidins, and isoflavones. Occasionally they can be found as aglycones but most flavonoids are attached to sugars (glycosides) [29].

In potatoes, one of the most abundant flavonoids is catechin, ranging between 0 and 204 mg/100 g dry weight [36,47,48,49,50]. Flavonols like quercetin and kaempferol rutinose are also present in potato tubers [3,7,42,51,52]. Some authors have also reported the presence of rutin [3,7,42,53].

Flavonoids were more than 30 mg per 100 g fresh weight in white fleshed potatoes and this level is nearly doubled in red and purple fleshed potatoes as a result of anthocyanins [54]. The color of red and purple potatoes is derived from anthocyanins [55]. Anthocyanins are a sub-class of pigmented flavonoids. The most common anthocyanidins (the deglycosylated forms of anthocyanins) present in potatoes are malvidin, petunidin, delphinidin, and peonidin in purple tubers and pelargonidin in red ones [48,56]. In addition to this anthocyanin, aglycones, cyanidin, and petanin are also found in potatoes [51,57].

The levels of phenolic compounds in potatoes can vary greatly [29] depending on the color and variety of the potato cultivars [43]. Table 1 reviews the range of individual phenolic compound contents reported in the literature.

## 3. Effect of Harvesting, Post-Harvest, and Technological Processes on Phenolic Content

The amount of phenolic compounds and their stability are dependent on several factors such as agrotechnical processes, climatic conditions, ripeness during harvest, and post-harvest manipulations [63], as well as genotype, storage conditions after harvest, and processing and cooking methods [24,48,64,65,66,67,68]. Many polyphenols, particularly phenolic acids, are directly implicated in the response of plants to different types of stresses such as thermal stress, biotic stress, and injuries, and in their tolerance to exposure to UV rays and ozone [44]. These phytochemicals show antimicrobial properties by raising concentrations after pathogen infection and contribute to healing by lignification of damaged zones [69].

These biotic and abiotic factors can affect potatoes and may cause a serious economic problem in countries where potatoes are cultivated over large areas [1]. In the last decade, the effect of environmental conditions such as location on the phenolic content of potatoes has been widely studied [50,60,70,71]. During tuber development, environmental conditions may influence the phenylpropanoid pathway and the polyphenolic composition in potato tubers [60]. Higher chlorogenic acid levels were found in warm locations with regular periods of drought, in comparison with high-altitude locations that are beneficial for potato cultivation. Organically grown potatoes reported significantly higher levels of chlorogenic acids compared to conventional treatments [72]. On the other hand, these studies demonstrated that the genotype of a potato has more effect on the phenolic content than the location [50,70]. Navarre *et al.* [52] determined the phenolic and antioxidant capacity of different potato genotypes.

Although wild species or primitive germplasm reported the highest phenolic content, commercial/industrial cultivars are preferred because they have the agronomic characteristics to be economically practical and could easily and more commonly be planted. Moreover, it is known that the most noteworthy phenolic genotypes were colored-flesh potatoes; however, consumer choice is for white-flesh potatoes, so they are the most produced. This encourages new harvest and/or post-harvest treatments in order to improve the phenolic content.

For improving the phenolic content of potatoes, some implementations were used before and some after harvesting. Ngadze *et al.* [44] reported that calcium contributes to increase caffeic and chlorogenic acid in potatoes. Furthermore, calcium soil amendment also improved the concentration of polyphenol oxidase (PPO) and peroxidase (POD) enzymes, which are involved in the phenolics and total soluble phenols metabolism. Several authors defined the role of Ca^2+^ in phenolic metabolism; Castañeda & Pérez [73] found that foliar application of 10 mM of CaCl_2_ increased phenylalanine activity and resulted in the accumulation of phenols [44]. Other authors used a curing treatment (10 days at 16 °C) immediately after harvest, obtaining a high phenolic content and PPO activity in potato flesh; moreover, they enhanced the fresh-cut color and the sensory qualities after cutting [69].

After harvest, storage conditions also play an important role in phenolic contents. While Singh & Saldaña [38] indicated that phenolic compounds may degrade during storage conditions, many other studies reported that cold storage (∼4 °C) of potatoes caused an increase in the phenolic content or kept it constant [40,61,68]. However, as described by Andre *et al.* [74], high storage temperatures (10 °C) decreased the phenolic contents or had no effect. On the other hand, Külen *et al.* [40] demonstrated that storage time is also very important for phenolic compounds. They found that the total phenolic content (TPC) of potatoes was high at harvest, declined after two months of cold storage, increased after four months of cold storage, and finally increased to almost harvest level after seven months of cold storage. The results of Blessington *et al.* [47] were in accordance with their data, showing that TPC decreased slightly after four months of cold storage in the ‘Russet Burbank‘ variety during cold storage. Further detailed studies are required to clarify the effects of cold storage on individual potato clones.

In addition to the harvest and post-harvest condition, cooking processes should be considered as well. The chemical, physical, and enzyme modifications that were produced during cooking will change the antioxidant capacity and digestibility of potatoes, which later influences the bioavailability of phytochemicals to the postprandial glycemic response of the human body [75]. In a study of Mulinacci *et al.* [76], the contents of phenolic acids, glycoalkaloids, and anthocyanins are measured in fresh and also in processed (boiling and microwave cooking) potatoes from three cultivars (Vitelotte Noire, Highland Burgundy Red with pigmented flesh, and Kennebec with white pulp). Different from the literature data, it is indicated that the heating treatment did not cause any changes in phenolic acids content except for a small decrease in anthocyanins. Also, Perla *et al.* [54] studied some potato cooking methods such as boiling, baking, and microwaving on phenolic compounds in five cultivars with different skin and flesh colors after six months of storage. The level of phenolic compounds was reduced by the three cooking methods but boiling minimized these losses.

Briefly, the cultivar, stage of maturity of the tuber, cooking temperature and time, and presence of water or moisture during cooking all strongly affect the loss of phenolic compounds in potatoes.

## 4. Extraction and Determination Methods for Phenolics in Potato

During the extraction of antioxidant compounds from plant materials, the selection of the appropriate extraction conditions represents a crucial point [18]. During the extraction, the distribution of phenolic compounds in extraction solvents should be made to reach the appropriate distribution coefficient [75]. To extract phenolic compounds from potatoes, the most common method is solid–liquid extraction [18,27,76]. Commonly, the extraction solvents used for potato phenolics are methanol, ethanol, and aqueous alcohol mixtures [46,77,78,79]. However, these techniques require a long extraction time and result in low yields [38]. Therefore, other modern extraction and isolation techniques have been applied as alternative techniques for potato phenolic extraction. Ultrasound-assisted extraction (UAE), microwave-assisted extraction (MAE), and pressurized liquid extraction (PLE) are a few of these modern techniques [45,62,80,81,82] (Table 2).

Sonication or ultrasound-assisted extraction (UAE) is a simple and efficient green technology for the extraction of phenolic compounds that could be used as an alternative to conventional shaking and warming steps. Sonication permits a greater penetration of the solvent into the sample matrix, increasing the contact surface area between the solid and liquid phase; as a result, the solute quickly diffuses from the solid phase into the solvent, improving the extraction yield [55,89]. Also, UAE can provide the possibility for improved extraction of heat-sensitive bioactives and food components at lower processing temperatures [90].

The use of microwave-assisted (MAE) extraction for food bioactives has increased significantly in recent years [82,91,92,93]. In MAE, the direct effect of microwaves on molecules by ionic conduction and dipole rotation permits an increase of temperature, and because of that a high recovery of bioactive compounds [94]. MAE is a fast, selective, and energy-saving method and requires lower amounts of solvents when compared to conventional heating methods [82,95].

The pressurized liquid extraction (PLE) of phenolics has also been recommended as a green extraction technique for antioxidants in potato peel [38,45]. This technology uses high pressures to maintain water at temperatures between 100 and 374 °C; however, it requires complex and high-cost equipment in large-scale industrial extractions [82]. Nevertheless, the use of high temperatures improved the mass transfer and extraction rates, thus PLE generally requires shorter extraction times and a lower consumption of organic solvents than conventional techniques [45,96].

After extraction, even though the analysis of phenolic compounds is very challenging due to the extensive diversity and reactivity of these compounds, novel separation and detection methods, such as hyphenated techniques of high-performance liquid chromatography (HPLC) with mass spectrometry (MS), ultraviolet-visible light (UV-Vis), or nuclear magnetic resonance (NMR) spectroscopy can be used successfully [87,97]. For the determination of total phenolic compounds in the potato tuber and peel, the most commonly used method is the Folin-Ciocalteu method [70,74,98]. On the other hand, among all these methods HPLC is the most frequently used for the identification of individual phenolic compounds present in the potato [13,18,31,38,80]. This analytical technique acquires a high degree of versatility not found in other chromatographic systems and allows for separating a wide variety of chemical mixtures [52].

## 5. Use of Potato Peel Extract as an Antioxidant

In the last decade, there has been increasing attention given to new sources of natural antioxidant phytochemicals as a result of their potential health benefits, in addition to their functional properties in traditionally commercialized products such as preserving color and flavor and hence improving shelf life [99,100,101].

Lipid oxidation is one of the most important causes of food quality deterioration; it generates off odors and off flavors, decreases shelf life, alters texture and color, and decreases the nutritional value of food [102]. Countless methods have been introduced to control the rate and extent of lipid oxidation in foods, but the addition of antioxidants is one of the most effective. Antioxidants have become a crucial group of food additives due to their ability to extend the shelf life of foods without any adverse effect on their sensory or nutritional qualities [32]. Generally synthetic antioxidants such as butylated hydroxytoluene (BHT) and butylated hydroxyanisole (BHA) are used to control oxidation, but these synthetic antioxidants are known to have carcinogenic and toxic effects on humans [16]. Therefore, the importance of replacing synthetic antioxidants with natural ingredients has increased significantly [19].

On the other hand, byproducts of food processing are a low-cost raw material for the extraction of healthy compounds such as dietary fiber, natural antioxidants, and natural food additives [103]. Also, fruit and vegetable waste and byproducts are discarded frequently at a cost to the manufacturer. Hence, use of the waste as a source of polyphenols may be of noticeable economic benefit to food processors [101,104]. In addition to this, since the concentration of phenolic compounds is higher in the peel than in the potato tuber, researchers generally used peels instead of using the whole potato for natural food additives [18,19,43].

A number of studies investigated the antioxidant effects of potato polyphenols (Table 3). Most of them searched for the effect on different oils [16,19,87]. Koduvayur Habeebullah *et al.* [16] used potato peel extract for fish oil and oil-in-water emulsions and their results showed that ‘Sava‘ variety potato peel extract is highly efficient at reducing lipid peroxidation. In another study performed by Amado *et al.* [18], ethanolic extract of potato peel waste was used to evaluate the ability to limit oil oxidation and, according to the results, extracts (obtained by extraction with a medium or high ethanol concentration) were able to stabilize soybean oil under accelerated oxidation conditions, minimizing peroxide (PV), anisidine (AV), and totox values (TV) at high temperature.

There are some studies that compare the antioxidant activity of potato peel extract (PPE) with commercial antioxidants. Kanatt *et al.* [43] found that the antioxidant activity of PPE is comparable to butylated hydroxytoluene (BHT). Also, Mohdaly *et al.* [87] estimated the antioxidant effect of potato peel by measuring both primary (hydroperoxides) and secondary oxidation products, and comparing them with sesame cakes and sugar beet pulp in terms of the effect on sunflower oil. The results showed that potato peels displayed more antioxidative effect than sesame cake and sugar beet pulp, performing as well as synthetic antioxidants (BHT and BHA). Albishi *et al.* [8] also found that Russet potato peel was more effective in inhibiting the formation of TBARS than BHA.

Some researchers used potato peels to limit the lipid oxidation in meat [8,9,43]. Kanatt *et al.* [43] added potato peel extract to meat before irradiation; the extract was able to retard lipid peroxidation without affecting its flavor/aroma, thereby improving its storage quality. In a study performed by Habeebullah *et al.* [9], potato peel was used in minced mackerel, a fatty fish particularly high in n-3 PUFA, as a natural antioxidant source for retarding lipid and protein oxidation [105]. The results suggest that ethanol extracts of potato peel can be used as a natural additive to prevent lipid and protein oxidation in chilled storage. Albishi *et al.* [8] also indicated that potato peel extracts were efficient in inhibiting the oxidation of cooked salmon; in fact, the control samples showed high TBARS values after seven days of storage. On the other hand, more studies are needed to support this hypothesis and extend the utilization of these extracts in foods (such as meat and fish products) where a complex mixture of proteins, lipids, pro-oxidants, and endogenous antioxidants are present.

Unfortunately, in the case of potato extracts, the recovery of phenolic compounds may reveal a problem because toxic glycoalkaloids might be concentrated during processing [16]. Glycoalkaloids are natural compounds produced in potatoes during germination that may have both adverse and beneficial effects [3,106]. These compounds can cause death at concentrations >330 mg/kg sample [107] but, depending on their concentration, they can also have positive effects (e.g., anti-carcinogenic effect against a series of human cancer cells *in vitro*) [108]. Because of that, it is very important to check the presence of these compounds in the extracts.

## 6. Health Benefits of the Potato

There is a growing interest in food-based approaches for chronic disease prevention [109]. Potatoes also gained increasing attention as a source of nutrients and bioactive phytochemicals [41,68,110,111]. Phenolic compounds have antioxidant activity and other characteristics that could promote health. A number of studies investigated these antioxidant, antiproliferative, and anticancer effects of potato polyphenols [84,112,113]. Table 4 shows some of these health effects of potatoes *in vivo* and *in vitro*.

Because of their potential health benefits, phenolic acids, among all phenolic compounds, have raised great interest [31]. Plazas *et al.* [114], in a study with eggplant, which is another Solanaceae plant with high chlorogenic acid content, indicated that chlorogenic acid presents many beneficial properties for human health, such as antioxidant, anticarcinogenic, anti-inflammatory, analgesic, antimicrobial, neuroprotective, and cardioprotective effects. Chlorogenic acid, which is widely found in potato samples, produced an increase of insulin sensitivity, decreased the gut glucose absorption, and prevented gluconeogenesis [115,116]. Singh *et al.* [117] demonstrated that chlorogenic acid could mitigate oxidative stress effects in streptozotocin-induced diabetic rats [13]. Moreover, chlorogenic acid demonstrates an antiproliferative activity in several cancer cells [118].

The antioxidant activity of phenolic compounds is essentially due to their redox properties [43,119]. Oxidative stress can induce oxidative damage to proteins, DNA, and lipids, resulting in an increased risk of cancer and cardiovascular disease. Adequate amounts of food phenols as antioxidants need to be consumed to inhibit or slow the oxidative damage induced by free radicals [28]. This explains the huge volume of scientific work aiming to connect diets rich in natural antioxidants with a decreased rate of degenerative disease [120].

Antioxidant activity in potato tubers has been extensively reported [84,113]. Pigmented potato genotypes (mainly cultivars with purple and red flesh), as compared to those with white and yellow flesh, have been shown to contain significantly higher levels of antioxidants [40,68]. Kaspar *et al.* [112] investigated the influence of pigmented potato consumption on oxidative stress and inflammatory damage in men for six weeks. The results showed that the consumption of yellow- and purple-fleshed potatoes reduced inflammation and DNA damage. The concentrations of C-reactive protein (a biomarker for disease progression) in plasma decreased according to the increase of consumption of potatoes that contain high amounts of anthocyanins [112]. Animal studies showed similar results. The consumption of purple potato flakes enhanced the antioxidant potential in the serum and liver of cholesterol-fed rats thanks to the enhancement of the expression of some hepatic antioxidant enzymes [59]. Moreover, red potato flakes enhanced the hepatic superoxide dismutase mRNA in rats, improving the antioxidant system [30,59].

A correlation between polyphenol intake and reduced incidence of some diseases has been noticed in several studies; however, their positive effects could not be assigned only to their antioxidant properties. In fact, the health benefits of polyphenols are assigned to some “non-antioxidant” complex activities that could not be related to the free radical inhibition. According to the recent literature, potato polyphenols may be used also for some non-antioxidant beneficial health effects [115]. For instance, potato extracts inhibited breast [49] and colon cancer cell proliferation [113], also showing pro-apoptotic properties in the latter case [121]. Singh, Kamath, & Rajini [119] studied the antihyperglycemic effect of potato peel in experimental rats. Other researchers reported that a freeze-dried powder of potato peel caused a notable decrease in blood glucose levels and effectively reduced diabetic change in rats [37,99].

Antioxidants produce cancer cell inhibition. Thompson *et al.* [111] fed rats with potatoes and investigated their role in breast cancer risk. They reported that rats fed on the ′Mountain Rose′ cultivar had a reduced cancer incidence (with evidence of a dose-dependent effect). In addition, lymph node carcinoma of the prostate and prostate cancer-3 prostate cancer cell proliferation have been prevented using an extract of colored potato and an anthocyanin-rich fraction [30,122]. Moreover, potato anthocyanin compounds were toxic to human stomach cancer cells and prevented the growth of benzopyrene-induced stomach cancer in mice [113,123]. Madiwale *et al.* [61] investigated the effects of potato antioxidants on HCT-116 and HT-29 colon cancer cell lines and found that the antiproliferative and pro-apoptotic activities were suppressed. They also confirmed that suppressing proliferation and elevating apoptosis in early and advanced human colon cancer cell lines increased when purple-fleshed potatoes were used.

Numerous phenolic compounds were analyzed to find their antioxidant and/or anticancer activity. Many studies correlated the antiproliferative and antioxidant activity, and demonstrated that oxidative mechanisms can affect the proliferation of cancer cells.

Usually, but not always, extracts with high phenolic content displayed higher antioxidant and cytotoxic activities [121]. Therefore, the potato could be an ideal source of health-promoting phytochemicals considering its high level of consumption all over the world. Nevertheless, the effects of phenolic compounds of the potato on health are dependent on the diversity of polyphenols classes that they contain [51]. Therefore, further investigation in this area is required.

## 7. Conclusions

Potatoes, which have an important place in human life, contain a wide variety of phenolic compounds. Although the phenolic content and antioxidant capacity of potatoes are lower than in some other plants, because of their high consumption rates they may promote higher phenolic and antioxidant intake. In addition, potato peels as a byproduct of potato processing are available in large amounts and, since peels have more phenolic compounds than tubers, these compounds could be used in food and non-food applications.

## Figures and Tables

**Table 1:** Concentration levels of the main phenolic compounds in potatoes.

**Phenolic Classes**	**Phenolic Compounds**	**Range (mg/100 g Dry Extract)**	**References**
*Phenolic acids*	chlorogenic acid	27.6	[43]
		100.0–220.0	[53]
		17.4–1274.6	[51]
		47.0–283.0	[49]
		17.3–1468.1	[36]
		21.0–40.0	[58]
		60.0–292.0	[17]
		0.2–2193.0	[3]
	caffeic acid	0.1–0.2	[53]
		5.0–50.0	[49]
		1.1–172.4	[36]
		2.0–6.9	[58]
		0–41.6	[3]
	coumaric acid	0–9.2	[49]
		0–1.6	[36]
	protocatechuic acid	0–7.6	[36]
	vanillic acid	0–22.4	[36]
	ferulic acid	0.6–9.0	[49]
		0–3.9	[36]
		0–1.4	[3]
	cryptochlorogenic acid	16.0–27.0	[53]
		3.1–163.3	[36]
		8.0–59.0	[17]
		0.1–168.3	[3]
	neochlorogenic acid	2.9–9.9	[53]
		49.2–91.2	[36]
		0.5–1.5	[58]
		3.0–11.0	[17]
		0.1–87.6	[3]
	gallic acid	0–1.0	[36]
	*p*-hydroxybenzoic acid	0–7.8	[36]
*Flavonols*	rutin	0.5–2.6	[53]
		0.6–1.3	[17]
		0–12.2	[3]
	kaempferol rutinose	0.5–1.7	[17]
	quercetin-3-*o*-glu-rut	2.5	[53]
*Flavan-3-ols*	catechin	43.0–204.0	[49]
		0–1.5	[36]
		0–1.4	[3]
*Anthocyanidins*	anthocyanins	1.4–163.3	[51]
		87.0	[59]
		953.8–1630.3	[60]
		21.0–109.0	[56]
**Phenolic Classes**	**Phenolic Compounds**	**Range (mg/100 g Fresh Product)**	**References**
*Phenolic acids*	chlorogenic acid	1.4–12.1	[39]
		0.9–27.0	[31]
		0.4–34.0	[41]
		0.4–30.1	[61]
		8.7–28.6	[38]
	caffeic acid	0–1.2	[41]
		0.6–10.2	[61]
		5.2–12.2	[38]
	coumaric acid	0.8–6.5	[38]
	protocatechuic acid	0.2–0.5	[31]
		6.1–10.3	[62]
		1.9–2.0	[38]
	vanillic acid	0.6	[31]
	ferulic acid	0.1	[31]
		0–0.1	[61]
		1.5–4.9	[38]
	syringic acid	0.2–0.5	[31]
		0.9–1.7	[38]
	*p*-coumaric acid	0.2–3.0	[31]
	sinapic acid	0.3–0.9	[31]
		0–0.4	[61]
	gallic acid	0.5–0.6	[38]

**Table 2:** Overview of the extraction systems and analytical methods used for phenolic compounds determination in potatoes.

Extraction System	Analytical Technique	Potato Cultivar	Phenolic Compounds Described	References
Solid-liquid extraction	HPLC-DAD	‘Kufri chandromukhi’	Chlorogenic acid, caffeic acid, gallic acid	[43]
	HPLC UV-Vis	9 italian cultivars (‘Agata‘, ‘Primura‘, ‘Arinda‘, ‘Merit‘, ‘Marabel‘, ‘Jelli‘, ‘Frinka‘, ‘Sponta‘, ‘Agria‘)	Chlorogenic acid	[39]
	HPLC-MS	‘Ranger Russet’ ‘Norkotah Russet’	Neochlorogenic acid, chlorogenic acid, caffeic acid, quercetin-3-*o*-glu-rut, rutin, kaempferol-3-*o*-rutinoside, cryptochlorogenic acid, quinic acid	[53]
	HPLC-DAD, HPLC-MS, HPLC-FLD	23 Native Andean cultivars	Chlorogenic acid, neochlorogenic acid, cryptochlorogenic acid, caffeic acid, protocatechuic acid, vanillic acid, ferulic acid, petanin, rutin, kaempferol-3-*o-*rutinoside	[51]
	HPLC-DAD	320 specialty potato genotypes	Chlorogenic acid, caffeic acid, gallic acid, catechin	[40]
	Not cited	‘Russet Burbank’	Chlorogenic acid, ferulic acid, vanillic acid, caffeic acid, benzoic acid	[83]
	HPLC-MS	‘Jasim’, ‘Atlantic’, ‘Jawan’, ‘Superior’, ‘Jopung’	Chlorogenic acid, caffeic acid, ferulic acid, *p*-coumaric acid, trans-cinnamic acid	[41]
	HPLC-DAD	‘Nicola’, ‘Sieglinde F’, ‘Isci 4052’, ‘Isci 67’	Chlorogenic acid, caffeic acid, ferulic acid, catechin	[49]
	HPLC	Not cited (Indian cultivar)	Gallic acid, caffeic acid, chlorogenic acid, protocatechuic acid	[84]
	HPLC-DAD	13 native Andean genotypes	Neochlorogenic acid, cryptochlorogenic acid, chlorogenic acid, kaempferol-3-*o*-rutinoside, quercetin	[60]
	HPLC	‘Karlena’	Gallic acid, neochlorogenic acid, protocatechuic acid, catechin, cryptochlorogenic acid, chlorogenic acid, vanillic acid, caffeic acid, ferulic acid, *p*-coumaric acid	[36]
	HPLC UV-Vis	‘Siecle’, ‘Purple Majesty’, ‘Dakota pearl’, ‘FL 1533’, ‘Vivaldi’, ‘Yukon gold’	Chlorogenic acid, caffeic acid	[13]
	HPLC-DAD, HPLC-MS	‘Goldrosh’, ‘Nordonna’, ‘Dakota Pearl’, ‘Norkotah’, ‘Red Nordland’, ‘Sangre’, ‘Viking’, ‘Dark Red Nordland’	Chlorogenic acid, caffeic acid, gallic acid, ferulic acid, catechin, *p*-coumaric acid, *o*-coumaric acid	[46]
	HPLC-DAD	8 cultivars	Chlorogenic acid, caffeic acid, epicatechin, *p*-coumaric acid, vanillic acid, quercetin	[47]
	HPLC-DAD	‘Sava’, ‘Bintje’	Protocatechuic acid, gentisic acid, gallic acid, chlorogenic acid, salicylic acid, caffeic acid, ferulic acid, *p*-coumaric acid	[19]
	HPLC-DAD-MS	‘Bintje’, ‘Piccolo’, ‘Purple Majesty’	Chlorogenic acid, neochlorogenic acid, cryptochlorogenic acid, kaempferol rutinose, rutin	[17]
	HPLC-DAD/APCI-MS	16 cultivars	Chlorogenic acid, caffeic acid, 3-*o*-caffeoylquinic acid, 1-*o*-caffeoylquinic acid	[77]
	HPLC-DAD-MS	13 Italian cultivars	5-*o*-caffeoylquinic acid, 4-*o*-caffeoylquinic acid, 3-*o*-caffeoylquinic acid, ferulic acid, anthocyanins	[66]
	UPLC-MS	‘Purple Majesty’, ‘Yukon gold’, ‘Atlantic’	Chlorogenic acid, caffeic acid, ferulic acid, sinapic acid	[61]
	HPLC-DAD-MS	50 cultivars	Chlorogenic acid, rutin, kaempferol-3-rutinose	[52]
	UPLC-DAD	‘Vitelotte’, ‘Luminella’, ‘Charlotte’, ‘Bintje’	Chlorogenic acid, neochlorogenic acid, cryptochlorogenic acid, caffeic acid, ferulic acid, *p*-coumaric acid, syringic acid, vanillic acid, catechin, rutin, kaempferol-3-*o*-rutinoside	[3]
	HPLC-DAD	‘Sava’	Gallic acid, protocatechuic acid, gentisic acid, chlorogenic acid, vanillic acid, syringic acid, caffeic acid, salicylic acid, *p*-coumaric acid, ferulic acid	[9]
	HPLC-DAD	Not cited	Chlorogenic acid, neochlorogenic acid, cryptochlorogenic acid, coumaric acid, genistin, quercetin-3-β-d-galactoside, naringin ,naringenin, luteolin, genistein, kaempferol, flavan-3-ol	[85]
	UPLC-MS	Not cited	chlorogenic acid, quinic acid, caffeic acid, methyl caffeate	[86]
	HPLC-DAD-MS	15 Colombian cultivars	Chlorogenic acid, neochlorogenic acid, cryptochlorogenic acid, caffeic acid	[76]
	HPLC UV	‘Agria’	Chlorogenic acid, ferulic acid, gallic acid	[18]
	HPLC UV	‘Valfi’, ‘Blaue Elise’, ‘Bore Valley’, ‘Blue Cango’	Chlorogenic acid, caffeic acid, ferulic acid, coumaric acid, cryptochlorogenic acid, neochlorogenic acid, *p*-coumaric acid	[27]
Ultrasound-assisted extraction	HPLC-DAD	‘Nicola’, ‘Timo’, ‘Siikli’, ‘Rosamund’, ‘Van Gogh’	Chlorogenic acid, caffeic acid, ferulic acid, sinapic acid, vanillic acid, syringic acid	[31]
	HPLC-DAD	‘Agria’	Protocatechuic acid, chlorogenic acid, neochlorogenic acid, cryptochlorogenic acid	[62]
	HPLC-DAD	20 potato cultivars	Chlorogenic acid, petunidin-3-glucoside chloride, pelargonidin-3-glucopyranoside	[81]
	HPLC-MS	‘Purple’, ‘Innovator’, ‘Russet’, ‘Yellow’	Chlorogenic acid, caffeic acid, *p*-coumaric acid, ferulic acid	[8]
	HPLC-DAD	‘Penta’, ‘Marcy’	Chlorogenic acid, caffeic acid, gallic acid, *p*-coumaric acid, ferulic acid	[15]
	HPLC-DAD	‘Diamond’	Chlorogenic acid, caffeic, 4-hydroxybenzoic, *p*-coumaric, and trans-*o*-hydroxycinnamic acids	[87]
	HPLC-DAD- -MS	‘Blue Bell’, ‘Melody’	Chlorogenic acid, caffeic acid, quinic acid, ferulic acid, cryptochlorogenic acid, rutin	[88]
	HPLC-MS	‘Russet’	Chlorogenic acid, caffeic acid, neochlorogenic acid	[42]
	RP-HPLC UV-DAD	‘BP1’	Chlorogenic acid, caffeic acid, ferulic acid	[44]
	HPLC-DAD	‘Netherlands #7’	Gallic acid, protocatechuic acid, chlorogenic acid	[69]
Microwave-assisted extraction	HPLC-UV	‘Red’	Chlorogenic acid, caffeic acid, gallic acid, protocatechuic acid, syringic acid, ferulic acid, coumaric acid	[38]
	HPLC-DAD	‘Calwhite’	Chlorogenic acid, caffeic acid, neochlorogenic acid, cryptochlorogenic acid, ferulic acid, *p*-coumaric acid	[82]
Pressurized liquid extraction (PLE) + solid-liquid extraction	HPLC-DAD	‘Lady Claire’	Caffeic acid	[45]
	HPLC-UV	‘Red’	Gallic, chlorogenic and syringic acid	[80]

HPLC: High Performance Liquid Chromatography; UPLC: Ultra Performance Liquid Chromatography; DAD: Diode Array Detector; UV: Ultraviolet detector; MS: mass spectrometer.

**Table 3:** Some materials in which potato peels were used as an antioxidant ingredient.

Food	Potato Type	Criteria	References
Processed Lamb Meat	Potato Peels (*Solanum tuberosum* cv. ‘Kufri chandramukhi‘)	TBARS and carbonyl content	[43]
Fish-Rapeseed Oil Mixture and in Oil-in-Water Emulsions	Potato peels (*Solanum tuberosum* cv. ‘Sava‘ and ‘Bintje‘)	Peroxide value, anisidine value, tocopherol concentration, and sensory evaluation	[19]
Soybean oil, sunflower oil	Potato peels (*Solanum tuberosum* cv. ‘Diamond‘)	Peroxide values, *p*-anisidine	[16]
Minced horse mackerel (*Trachurus trachurus*)	Potato Peels (*Solanum tuberosum*‘Sava‘ variety	Peroxide value, volatiles, carbonyl compounds, and protected against the loss of a-tocopherol and tryptophan and tyrosine residues	[9]
Ground Salmon	Potato peels and tubers (‘Purple‘, ‘Innovator‘, ‘Russet‘ and ‘Yellow‘)	TBARS	[8]
Sunflower oil	Potato peels (*Solanum tuberosum* cv. ‘Diamond‘)	Both primary (hydroperoxides) and secondary oxidation products	[87]
Soybean oil	Potato peel (Agria)	Peroxide, totox and *p*-anisidine values	[18]

TBARS, Thiobarbituric acid reactive substances.

**Table 4:** Health effects of potatoes.

Part of Potato	*in Vivo*/*in Vitro*	Subject	Effect	Disease	References
Potato flakes	*in vivo*	Male rats fed a high-cholesterol diet	Antioxidant effects	Oxidative stress	[59]
Extracts of peel and whole potatoes	*in vitro*	Human mammalian cancer cell (MCF-7)	Antioxidant activity; antiproliferative activity	Breast cancer	[49]
Potato peel extract	*in vitro*	Rat erytrocyte, Human erytrocyte membrane	Antioxidant effects	Oxidative damage	[84]
Whole potato	*in vitro*	Breast cancer cultures MCF-7 and MDA-MB-468	Anti-carcinogenic properties	Breast cancer	[68]
Whole potato	*in vivo*	20-day-old rats	Anticancer activity, antioxidant capacity	Breast cancer	[111]
Whole potato	*in vivo*	Free-living healthy men	Antioxidant effects, Anti-inflammatory activity	Oxidative stress and inflammation biomarkers	[112]
Whole potato extracts	*in vitro*	Human Colon Cancer Cell Lines	Antioxidant activity, anticancer properties	Colon cancer	[61]
Potato peel tuber and granule	*in vitro*	HepG2 liver cells	Antioxidant effects, and neuroprotective activities	Liver LDL (Low-density lipoprotein ) cholesterol uptake and protection of cortical neurons from cell death	[81]
Whole potato	*in vitro*	Human colon cancer cell lines	Antioxidant activity, antiproliferative and pro-apoptotic properties	Colon cancer	[113]
